# The prevalence of primary lung cancer among women with previous personal and/or family history of cancer in an integrated health system

**DOI:** 10.1016/j.xjon.2026.101823

**Published:** 2026-04-17

**Authors:** Carissa A. Villanueva, Tyler Chervo, Andrea Gochi, Hyunjee Kwak, Lue-Yen Tucker, Jeffrey B. Velotta

**Affiliations:** a Division of General Surgery, Department of Surgery, UCSF East Bay, Oakland, Calif; b Division of Research, Kaiser Permanente Northern California, Pleasanton, Calif; c Department of Clinical Science, Kaiser Permanente Bernard J. Tyson School of Medicine, Pasadena, Calif; d Division of Thoracic Surgery, Department of Surgery, Kaiser Permanente Oakland, Oakland, Calif

**Keywords:** lung cancer, lung cancer screening, female lung cancer

## Abstract

**Objective:**

To evaluate whether personal and/or family history of cancer increases the prevalence of new primary lung cancer in adult women, regardless of smoking status.

**Methods:**

A retrospective review was performed using the data from Kaiser Permanente Northern California region electronic health record between January 1, 2010, and December 31, 2022. We included all adult women without a previous personal history of lung cancer and with at least 1 year of membership during the study period. We estimated the prevalence ratio (PR) of new primary lung cancer among people with a personal or family history of cancer using targeted maximum likelihood estimation adjusting for smoking status, race/ethnicity, age, Charlson comorbidity index, and Neighborhood Deprivation Index.

**Results:**

We identified 2.8 million patients who met the selection criteria. A previous personal history of any cancer was associated with an 88% increased prevalence of lung cancer (PR, 1.88; 95% CI, 1.84-1.92); a family history of any cancer also was associated with increased prevalence (PR, 1.23; 95% CI, 1.20-1.26). Specifically, a previous personal history of breast, cervical, uterine, or ovarian cancers was also associated with increased prevalence.

**Conclusions:**

Personal history of previous malignancy and family history of malignancy were associated with increased prevalence of future lung cancer in our population. It suggests that smoking status alone in our population is not sufficient for gauging clinical suspicion of new primary lung cancer. Reevaluation and expansion of guidelines of screening criteria for use of lung cancer screening tools may be prudent.

**Figure:**
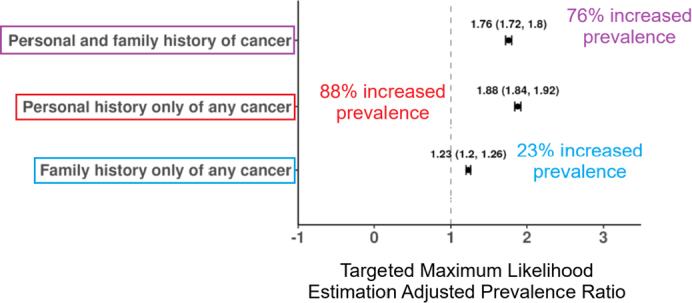
Prevalence ratios for those with a personal/family history of cancer versus without.

**Central Message**

Smoking status alone is likely not sufficient for evaluating use of lung cancer screening in women. Personal history of cancer and family history of cancer should also be taken into account.

**Perspective**

Current lung cancer screening guidelines focus only on smoking status. Recent data suggest this alone may be insufficient, especially in younger women. No study to date has looked at history of cancer and relative risk of future lung cancer. Our results support personal and family history of cancer as risk factors, regardless of smoking status. We hope to use this to develop more nuanced risk models.

Although breast cancer is the most common malignancy in female patients, lung cancer has the greatest mortality rate in the United States.^1^ Smoking has long been recognized as an independent risk factor for the development of lung cancer. Current US Preventive Services Task Force new recommendations for lung cancer screening include yearly low-dose computed tomography (LDCT) scanning for adults aged 50 to 80 years, current smokers, or with a 20 pack-year smoking history who have quit smoking within the past 15 years. This screening method has led to earlier diagnosis and treatment, resulting in reduced mortality from lung cancer.^2^^,^^3^ Traditionally, lung cancer prevalence is increased in older, male smokers.

However, recent studies have found that a family history (particularly in a maternal relative) of lung cancer is a risk factor for primary lung cancer among never-smokers and younger adults.^4^ Further supporting this was the recent Taiwan Lung Cancer Screening in Never-Smoker Trial, in which the authors found similar results: among nonsmokers, a family history of lung cancer was the greatest risk factor for the detection of lung cancer.^5^ The authors of another study identified that a previous diagnosis of head and neck cancer was significantly associated with the development of subsequent primary lung cancer, even among individuals who had never smoked.^6^ In addition, this study observed that women with a previous history of breast cancer have significantly greater risk of developing a subsequent primary lung cancer compared with the general population.^6^ The impact on outcomes in this patient population is not yet clearly understood, but second primary cancers have previously been shown to have a negative impact on survival.^7^ To date, no studies have specifically looked at the relative risk of individuals with a previous history of cancer of developing a second, new primary lung cancer stratified by the specific type of cancer they had (ie, previous personal history of breast cancer vs previous personal history of uterine cancer, etc). In addition, no guidelines exist for lung cancer screening in patients who may be at particularly high risk regardless of smoking status. Implementation of lung cancer screening in patients with a personal or family history of cancer and high-risk features for developing a subsequent primary lung cancer regardless of smoking status may allow for more prompt diagnosis and treatment. The goal of our study is to identify additional risk factors, including personal or family history of specific cancers, for the development of primary lung cancer in women. This would hopefully aid in the development of novel lung cancer screening risk models for other at-risk populations other than smoking history.

## Methods

### Human Subjects Protection

This study was approved by the Kaiser Permanente Northern California (KPNC) Institutional Review Board with a waiver of written informed consent. The institutional review board number is 2182779-4 and was approved on February 24, 2025.

### Setting

KPNC is a large, integrated health care system that serves more than 4.5 million members in Northern and Central California, including the San Francisco Bay Area.^8^ KPNC members receive the vast majority of their care at KPNC facilities, including cancer screening, diagnosis, and treatment. Because KPNC is both a payer and provider of health care services, our electronic health record has an excellent capture of events that occur within the health care system. The characteristics of KPNC members are highly representative of the population in counties in which KPNC has a physical presence^9^ and includes patients covered by Medicare, Medicaid, and commercial insurance.

### Study Objectives and Variable Definitions

The primary purpose of our study was to estimate the adjusted prevalence ratio (PR) of new primary lung cancer among women with a personal or family history of cancer relative to those without those histories. To accomplish this, we performed a retrospective cross-sectional study using data from KPNC region electronic health record between January 1, 2010, and December 31, 2022. We included all adult women (age 18 or older) without a personal history of lung cancer and with at least 1 year of KPNC membership during the study period. Kaiser Permanente maintains a Surveillance, Epidemiology, and End Results Program–compliant cancer registry that was used to identify cases of lung cancer for this study.

We also ascertained a personal history of cervical, uterine, breast, and ovarian cancer using a combination of *The International Classification of Diseases*, *Ninth*/*Tenth Revision* (ICD-9/10-CM), *Clinical Modification*, diagnosis codes and the *International Classification of Diseases for Oncology*, *Third Edition*, from the KPNC Cancer Registry. Family history of lung cancer and breast cancer were also assessed using ICD-9 and ICD-10 codes. A personal history of any cancer was assessed using a combination of ICD-9 and ICD-10 codes as well as having an entry for any cancer in the KPNC cancer registry. Family history of lung and breast cancer was assessed using ICD-9 and ICD-10 codes. Personal history of any cancer was identified using a combination of ICD-9/10 diagnosis codes and entries in the KPNC Cancer Registry, and family history of any cancer was similarly ascertained using ICD-9 and ICD-10 codes. A list of all ICD-9, ICD-10, and *International Classification of Diseases for Oncology*, *Third Edition*, codes used in this study can be seen in Table E1. To validate our ascertainment of primary lung cancer cases, clinical team members reviewed the electronic health records of a sample of patients to confirm that their tumors were primary lung cancer cases. The personal and family histories of these cancers were the primary exposures for our study.

We additionally ascertained the following covariates: age in years at first date of eligibility for the study, race/ethnicity, smoking history, Charlson comorbidity index score category (unknown, no visits in the past year, 0, 1, 2+), and body mass index (BMI) category. BMI category was categorized as “unknown,” “underweight” (BMI <18.5), “normal weight” (BMI 18.5-24.9), “overweight” (BMI 25-29.9), and “obese” (BMI ≥30). Information on smoking history was obtained from a questionnaire that is routinely administered during health care visits at KPNC. All data for covariates were obtained from the KPNC electronic health record. A personal/family history of cancer was assessed before the date of lung cancer diagnosis among people with the outcome or at any time during the study period for people without the outcome. All other variables were ascertained as close to the individual's date of first eligibility as possible.

### Statistical Methods

To estimate the adjusted PR of new lung cancer among people with a personal or family history of cancer relative to those who do not, we used targeted maximum likelihood estimation (TMLE). TMLE is a highly flexible framework that produces doubly robust and statistically efficient estimates for a given target parameter.^10^ Our target parameter of interest for this study was the aforementioned covariate-adjusted PR. A major benefit of TMLE is its internal use of the Super Learner algorithm, which creates a weighted, cross-validated ensemble of specified candidate “learner” algorithms that minimizes the variance of the model output.^11^ The candidate learner library we considered included generalized linear regression models, least absolute shrinkage and selection operator regression, extreme gradient boosted decision trees (“XGBoost”), and a negative control learner that assigns everyone the same mean predicted value.

All TMLE models were adjusted for age, race/ethnicity, smoking history, Charlson comorbidity index score category, Neighborhood Deprivation Index, and BMI category. Models with specific cancers as the primary exposure (such as ovarian cancer) were adjusted for other specific types of cancers in their models. The models that had broader groups of cancer (such as a personal history of any cancer) did not adjust for specific cancers to prevent multicollinearity. The reference level for all models was individuals without the personal/family history of cancer of interest. Univariate analyses were conducted using χ^2^ tests for categorical variables and *t* tests for continuous variables. Data extraction was performed using SAS, version 9.4, and the analysis was done using the R programming language, version 4.3.1. The packages *tidyverse* and *tmle3* were used in the R analysis.

## Results

We identified 2,866,410 KPNC members who met selection criteria for this study, of whom 10,595 patients in this population were diagnosed with lung cancer within the study period (Table 1). Compared with women without lung cancer, those diagnosed with lung cancer were older (mean 66 [11]; mean 41 [18]; *P* < .001) and more likely to have a history of smoking and a greater comorbidity burden (21% vs 12%, *P* < .001, and ≥2 [12% vs 3.4%, *P* < .001]). Non-Hispanic White patients made up 44% of the entire cohort and accounted for 69% of all patients with lung cancer. Patients of Asian descent comprised the second greatest number of cases of lung cancer (13%) but were the third-largest ethnic group. Patients with lung cancer were significantly more likely to have a personal history of any cancer compared with those without lung cancer (19% vs 6.1%, *P* < .001) on univariate analysis.

**Table 1:** Characteristics of female Kaiser Permanente Northern California members with and without lung cancer, 2010-2022

Characteristics	Lung cancer, N = 10,528∗	No lung cancer, N = 2,855,018∗	*P* value†
Age at anchor date, y	66 (11)	41 (18)	<.001
Smoking status			<.001
Never	3015 (29%)	1,822,331 (64%)	
Former	3657 (35%)	287,861 (10%)	
Current	2723 (26%)	180,563 (6.3%)	
Unknown	1133 (11%)	564,263 (20%)	
Race/ethnicity			<.001
Non-Hispanic White	7248 (69%)	1,242,067 (44%)	
Black	883 (8.4%)	194,678 (6.8%)	
Asian	1353 (13%)	562,524 (20%)	
Hispanic, any race	950 (9.0%)	589,880 (21%)	
Unknown/other	94 (0.9%)	265,869 (9.3%)	
NDI quartile			<.001
Missing	19 (0.2%)	10,452 (0.4%)	
Q1	1695 (16%)	530,971 (19%)	
Q2	3441 (33%)	873,320 (31%)	
Q3	3152 (30%)	802,198 (28%)	
Q4	2221 (21%)	638,077 (22%)	
English as primary language	9944 (94%)	2,546,889 (89%)	<.001
Charlson comorbidity index score category			<.001
No visits in the past year	504 (4.8%)	355,146 (12%)	
0	4196 (40%)	1,955,077 (68%)	
1	2170 (21%)	345,068 (12%)	
≥2	1314 (12%)	102,326 (3.6%)	
Missing	2344 (22%)	97,401 (3.4%)	
Body mass index category			<.001
Underweight	409 (3.9%)	139,808 (4.9%)	
Normal weight	3777 (36%)	1,008,494 (35%)	
Overweight	2841 (27%)	626,852 (22%)	
Obese	3015 (29%)	736,576 (26%)	
Unknown	486 (4.6%)	343,288 (12%)	
Personal history of any cancer	1900 (18%)	174,536 (6.1%)	<.001
Personal history of breast cancer	1204 (11%)	92,323 (3.2%)	<.001
Personal history of uterine cancer	140 (1.3%)	15,869 (0.6%)	<.001
Personal history of ovarian cancer	57 (0.5%)	7810 (0.3%)	<.001
Personal history of cervical cancer	82 (0.8%)	8205 (0.3%)	<.001
Family history of breast cancer	612 (5.8%)	164,531 (5.8%)	.8
Family history of lung cancer	183 (1.7%)	12,673 (0.4%)	<.001

∗ Mean (SD); n (%).

† Welch 2-sample *t* test; Pearson χ^2^ test.

Among patients with lung cancer, breast cancer was the most common previous malignancy (11%), followed by uterine (1.3%), cervical (0.8%), and ovarian cancer (0.5%), with each occurring more frequently than in patients without lung cancer (all *P* < .001). Although the prevalence of family history of breast cancer was similar between groups, patients with lung cancer were significantly more likely to report a family history of lung cancer (1.7% vs 0.4%, *P* < .001).

In adjusted analyses using TMLE-estimated PR (Table 2), a personal history of any cancer was associated with an 88% increased prevalence of lung cancer (PR, 1.88; 95% CI, 1.84-1.92), whereas a family history of any cancer was associated with a more modest increase in prevalence (PR, 1.23; 95% CI, 1.20-1.26). Individuals with both a personal and family history of cancer had a PR 1.74 (95% CI, 1.70-1.78). When we examined specific cancer types, a personal history of breast cancer (PR 1.56; 95% CI, 1.48-1.63), cervical cancer (PR, 2.03; 95% CI, 1.95-2.10), uterine cancer (PR, 1.51; 95% CI, 1.43-1.60), and ovarian cancer (PR, 1.28; 95% CI, 1.22-1.34) were each associated with increased lung cancer prevalence. A family history of lung cancer was strongly associated with lung cancer prevalence (PR, 2.40; 95% CI, 2.30-2.50), whereas a family history of breast cancer was not (PR, 0.83; 95% CI, 0.77-0.89). All estimates are relative to a counterfactual where no patient had the cancer of interest and/or family history of cancer.

**Table 2:** Targeted maximum likelihood estimation–adjusted prevalence ratios of outcome lung cancer by personal/family history of any or individual cancers

Exposure	Estimate	Lower 95% CI	Upper 95% CI
Personal history only	1.88	1.84	1.92
Family history only	1.23	1.20	1.26
Personal and family history	1.74	1.70	1.78
Personal history of breast cancer	1.56	1.48	1.63
Personal history of cervical cancer	2.03	1.95	2.10
Personal history of uterine cancer	1.51	1.43	1.60
Personal history of ovarian cancer	1.28	1.22	1.34
Family history of lung cancer	2.40	2.30	2.50
Family history of breast cancer	0.83	0.77	0.89

All estimates are relative to a counterfactual where no one has the personal or family history of cancer denoted in the exposure. All results in this table were adjusted for age, race/ethnicity, Neighborhood Deprivation Index quartile, the patient's primary language being English, smoking history, Charlson comorbidity index score category, and body mass index category. Results for a personal/family history of cancer were also adjusted the other specific personal/family histories of cancer listed in the table (ie, the personal history of breast cancer model also adjusted for a personal history of cervical cancer, uterine cancer, ovarian cancer, etc). All estimates are relative to a counterfactual where no one has the cancer of interest.

## Discussion

Our study shows that having a personal history of any previous cancer, a family history of cancer, or both is associated with increased prevalence of a primary lung cancer, regardless of smoking status. Our analyses found that a previous personal history of breast, uterine, ovarian, or cervical cancer all are associated with increased prevalence of primary lung cancer. It suggests that smoking status alone is not sufficient for gauging clinical suspicion of new primary lung cancer. Reevaluation and expansion of guidelines of screening criteria for use of LDCT to include certain demographic of women with certain types of previous personal and/or family history of cancer may be prudent given the strong relationship seen in our data.

An important consideration in interpreting these findings is the role of routine oncologic surveillance imaging. Many cancer surveillance protocols include imaging the chest to evaluate for distant recurrence, which may lead to earlier detection of incident lung cancers and contribute to apparent stage migration among patients with a previous cancer diagnosis. Recent studies suggest that increased diagnostic intensity among cancer survivors, including surveillance imaging, may contribute to earlier detection of subsequent primary lung cancers.^6^^,^^7^ However, surveillance imaging is heterogeneous across cancer types, varies in duration, and often concludes after 5 years. Notably, our findings demonstrate elevated lung cancer prevalence across multiple previous cancer types, including those for which routine imaging of the chest is not uniformly recommended or may have already concluded. These findings suggest a potential increased risk beyond standard surveillance windows. These results further highlight a potential gap between existing cancer surveillance practices and long-term lung cancer risk.

Similar to our findings, previous studies have demonstrated survivors of cervical cancer to be at greater risk of developing future lung cancer.^12^, ^13^, ^14^, ^15^ Chaturvedi and colleagues found that survivors of cervical adenocarcinoma were still at high risk of future lung cancer despite cervical adenocarcinoma not being considered a cancer related to cigarette smoking. The authors postulated that other factors, such as human papillomavirus infection, may be associated with the development of lung cancer, a finding echoed by more recent research.^16^ Similarly, previous literature has shown that patients who have had previous uterine or ovarian cancers are at a greater risk of developing a second primary lung cancer.^16^^,^^17^

Many studies have demonstrated a relationship between having a personal history of breast cancer and developing lung cancer. Two meta-analyses involving more than 4 million patients demonstrated that patients with breast cancer have a significantly greater risk of developing a second primary lung cancer with a standardized incidence ratio of 1.25, with smoking and radiotherapy (only for ipsilateral lung cancer) being independent risk factors.^18^ A separate study looking at more than 6000 women in the Surveillance, Epidemiology, and End Results database found that patients with triple-negative breast cancer (TNBC) developed secondary primary lung cancer at an even greater incidence whereas patients with hormone receptor positive disease had rates not significantly increased compared with the general population.^19^ The authors postulate that, given overexpression of epidermal growth factor receptor in both TNBC and lung adenocarcinoma, this may be a common pathway in the 2 disease processes, which has also been demonstrated in the literature.^20^ Given the data showing association of future lung cancer in patients with a history of breast cancer, proponents have advocated for adopting dual cancer screening programs particularly for female patients at high risk (ie, TNBC).

Taken together, the associations we observed for both personal and family history of cancer highlight that lung cancer risk in women cannot be fully explained by smoking behavior alone. These findings suggest a layered risk profile, where both individual cancer history and familial aggregation contribute to susceptibility, underscoring the need for screening models that capture these additional dimensions. Large-scale analyses have consistently demonstrated that individuals with an affected first-degree relative have a significantly greater likelihood of developing lung cancer, with the risk further elevated when multiple relatives are affected or when the proband's diagnosis occurs at a younger age.^21^^,^^22^ Importantly, this excess risk is not uniform across populations. Studies show that familial risk is greater in Asian cohorts than in Western cohorts, with the strongest associations observed among women and never-smokers.^23^^,^^24^ These patterns highlight that familial clustering of lung cancer cannot be explained by shared smoking behaviors alone.

Mechanistic explanations for familial aggregation include inherited germline mutations such as epidermal growth factor receptor T790M, polymorphisms in DNA repair genes, and other susceptibility variants, which may heighten vulnerability to environmental carcinogens.^21^^,^^25^ More recently, prospective studies such as the TALENT trial in Taiwan have shown that never-smokers with a family history of lung cancer remain at heightened risk and may benefit from targeted LDCT screening.^5^

Despite recommendations from the medical community, lung cancer screening adherence remains low at around 4-6% whereas approximately 72% of eligible women adhere to screening mammography for breast cancer.^26^ Numerous studies have shown that a significant fraction of women undergoing screening mammography are also eligible for lung cancer screening.^27^, ^28^, ^29^ This low compliance in lung cancer screening is particularly worrisome for patients with a previous personal history of malignancy, as this is a risk factor for a new primary, future cancer.^30^ As our population ages and modern medicine improves with current cancer therapies, more patients will be at increased risk of an additional cancer after their initial malignancy has been treated.^31^ Despite this knowledge, there has largely not been many attempts to implement dual screening programs. However, given the relatively high compliance rate of women with screening mammograms, there is potential to use this as an avenue to increase lung cancer screening.

The Pink and Pearls Campaign was one of the first studies to address the feasibility of dual screening in female patients.^32^ In their pilot study, women received routine screening mammograms and LDCTs using the 2021 US Preventive Services Task Force criteria. They were able to diagnose 3 patients of the 63 enrolled and had 0 deaths. Among the 188 other eligible women that did not enroll, 7 were later diagnosed with lung cancer resulting in 5 deaths. This initiative was significant for attempting implement a dual breast and lung cancer screening program for female patients.

A follow-up prospective survey study of patients undergoing screening mammography showed that 87% of patients were interested in undergoing dual breast and lung cancer screening. Of those patients who did undergo both, 84% expressed that they would likely undergo dual screening again.^33^ Of note, primary patient concerns involved logistics including if same-day screening could be implemented as well as cost coverage by insurance. The authors acknowledged that although they demonstrated the feasibility of dual screening, nationwide implementation is offset by many challenges, given the variability of resources and patient populations at various institutions and represent important areas for future research.

The Pink and Pearls Campaign showed that patients are willing to be screened for lung cancer. The original initiative used only the most recent US Preventive Services Task Force criteria for including women for lung cancer screening. This study was of particular interest to us, as an interesting implementation of the Pink and Pearls Campaign could be to integrate data that our group and others have shown. By using a more nuanced stratification approach on the basis of previous personal history of cancer of the patient in addition to smoking status alone, it may be possible to increase lung cancer screening in women who are high risk, regardless of smoking status. There are multiple strengths to our study. We used a very large diverse patient population, and the integrated nature of our system allows more accurate ascertainment of study variables. In addition, because TMLE is a flexible analytic approach, our estimates did not rely on the same assumptions that many traditional modeling methods do. Limitations to our study include its retrospective and cross-sectional nature. Due to being cross-sectional, our study did not consider the time each patient in our study was enrolled in a KPNC health plan beyond the eligibility criteria for this study. As such, some individuals in our data set were at risk of the outcome for longer periods of time than other members, which may make our prevalence estimates an underestimate. Furthermore, despite our overall sample size being large, some of our exposure categories had a limited sample size. Although we believe our use of data-adaptive methods in our analysis reduces any potential biases that could arise from these smaller sample sizes, it is possible that some bias may remain in our estimates after adjustment. The current analysis also did not analyze certain cancers, including colon cancer, alone. Colon cancer as well as other cancers were included in our analysis (as a part of the “any cancer” group), but for feasibility purposes, we chose to analyze a discrete number of cancers (ie. breast, uterine, ovarian, and cervical) alone as a starting point for this data. In addition, we chose the aforementioned cancers because of their particular relevance to our population of interest in this particular study—women.

In upcoming studies, we would like to analyze other types of cancer alone, particularly colon cancer. Furthermore, there has been more evidence recently that younger women have been found to have a greater incidence of lung cancer compared with younger men in which smoking alone was not attributing.^4^ Although the overall incidence of lung cancer has been decreasing in the United States, the rate of decline is much less for female patients compared with their male counterparts.^34^ An additional future extension of our research includes the analysis of specifically Asian female nonsmokers, a subgroup known to be highly affected by lung cancer.^35^ Finally, another future goal would be to include men into our study population. We would like to create a similar analysis with the male population to explore whether previous personal history of cancer is a large risk factor for new primary lung cancer as we have seen in the female population. In conclusion, our study demonstrates that having a personal history of any previous cancer, a family history of any cancer, or both are associated with increased prevalence of a primary lung cancer in female patients, regardless of smoking status. We hope to continue our investigation further by analyzing risk factors in male nonsmokers and expand our list of specific types of cancers to colon, prostate, or others. Furthermore, a future analysis would focus specifically on Asian women and the subgroup ethnicities under that racial umbrella. Our ultimate goal is to define a more comprehensive and nuanced set of risk factors, in addition to the current guidelines, for clinicians to use when stratifying a patient's risk for lung cancer and for determining appropriate candidates for LDCT for lung cancer screening.

## Conflict of Interest Statement

The authors reported no conflicts of interest.

The *Journal* policy requires editors and reviewers to disclose conflicts of interest and to decline handling or reviewing manuscripts for which they may have a conflict of interest. The editors and reviewers of this article have no conflicts of interest.

## Figures and Tables

**Table E1:** *International Classification of Diseases* (ICD) codes used to identify a personal or family history of cancers considered in our study

History type	ICD version	Codes
Family history of any cancer	ICD-9	V16.0, V16.1 V16.2, V16.3 V16.4, V16.5, V16.6, V16.7, V16.8, V16.9
	ICD-10	Z80.0, Z80.1 Z80.2, Z80.3, Z80.4, Z80.5, Z80.6, Z80.7, Z80.8, Z80.9
Family history of breast cancer	ICD-9	V16.3
	ICD-10	Z80.3
Family history of lung cancer	ICD-9	V16.1
	ICD-10	Z80.1
Personal history of any cancer	ICD-9	V10.0, V10.1, V10.2, V10.3, V10.4, V10.5, V10.6, V10.7, V10.8, V10.9
	ICD-10	Z85
History of breast cancer	ICD-9	V10.3
	ICD-10	Z85.3
History of cervical cancer	ICD-9	V10.41
	ICD-10	Z85.1
History of ovarian cancer	ICD-9	V10.43
	ICD-10	Z85.43
History of uterine cancer	ICD-9	V10.42
	ICD-10	Z85.42
Outcome lung cancer	ICD-O-3	C340-C349
